# Dental Clinic Deserts in the US: Spatial Accessibility Analysis

**DOI:** 10.1001/jamanetworkopen.2024.51625

**Published:** 2024-12-23

**Authors:** Md. Shahinoor Rahman, Jeffrey C. Blossom, Ichiro Kawachi, Renuka Tipirneni, Hawazin W. Elani

**Affiliations:** 1 Department of Oral Health Policy and Epidemiology at the Harvard School of Dental Medicine, Boston, Massachusetts; 2 School of Public Health at LSU Health Sciences Center New Orleans, Louisiana; 3 Center for Geographic Analysis, Harvard University, Cambridge, Massachusetts; 4 Department of Social and Behavioral Sciences at the Harvard T.H. Chan School of Public Health, Boston, Massachusetts; 5 Divisions of General Medicine and Hospital Medicine, Department of Internal Medicine, University of Michigan, Ann Arbor; 6 Department of Health Policy and Management at the Harvard T.H. Chan School of Public Health, Boston, Massachusetts

## Abstract

**IMPORTANCE:**

Little is known about the spatial accessibility to dental clinics across the US.

**OBJECTIVE:**

To map the spatial accessibility of dental clinics nationally and to examine the characteristics of counties and US Census block groups with dental care shortage areas.

**DESIGN, SETTING, AND PARTICIPANTS:**

Cross-sectional study of US dental clinics in 2023 using data from the IQVIA national practitioners’ database, which includes 205 762 active dentists. Data were analyzed from November 2023 to April 2024.

**EXPOSURES:**

Socioeconomic characteristics of block groups and counties including rurality, area deprivation, racial and ethnic segregation, and uninsured population.

**MAIN OUTCOMES AND MEASURES:**

Enhanced 2-step floating catchment area method with a 30-minute drive time impedance was used to calculate the accessibility score to dental clinics at the block group level. The outcomes were dental clinic shortage areas and inequality in access to dental clinics.

**RESULTS:**

Nearly 1.7 million people in the US (0.5%) lacked access to dental clinics within a 30-minute drive. This included 0.9 million male (52.2%), 1.2 million White (71.0%), 52 636 Black (3.0%), and 176 885 Hispanic (10.2%) individuals. Approximately 24.7 million people (7.5%) lived in dental care shortage areas (defined as <1 dentist per 5000 population). There was a significant difference in spatial accessibility scores between rural and urban areas, with 1 dentist for every 3850 people in rural areas and 1 dentist for every 1470 people in urban areas. Additionally, there were 387 counties with significant disparities in access to dental clinics. Rural block groups (23.9 percentage points [pp]; 95% CI, 23.6-24.3), block groups with higher levels of Black (1.5 pp; 95% CI, 1.3-1.7) and Hispanic (4.5 pp; 95% CI, 4.3-4.8) segregation, and block groups with the highest levels of area deprivation (5.5 pp; 95% CI, 5.1-5.9) were more likely to experience dental care shortages compared with urban block groups and those with lower levels of segregation and area deprivation. Moreover, rural counties (11.3 pp; 95% CI, 8.9-13.7), counties with a high uninsured population (3.0 pp; 95% CI, 1.5-4.4), and counties with high levels of deprivation (5.8 pp; 95% CI, 2.1-9.5) were more likely to have inequality in access to dental clinics.

**CONCLUSIONS AND RELEVANCE:**

In this cross-sectional study of US dental clinics, there was geographic shortage and maldistribution of the dental workforce. These findings can support dental workforce planning efforts at the federal and state levels to encourage dentists to practice in underserved areas to reduce disparities in access to dental care.

## Key Points

**Question:**

What is the geographic distribution of spatial accessibility to dental clinics in the US, and what are the characteristics of counties and US Census block groups with dental care shortage areas?

**Findings:**

This cross-sectional study of 205 762 US dentists found that nearly 1.7 million people in the US did not have access to dental clinics within a 30-minute drive, and 24.7 million lived in dental care shortage areas. Rural block groups and those with high levels of segregation and socioeconomic deprivation were more likely to experience dental care shortages compared with urban block groups and those with low levels of segregation and socioeconomic deprivation.

**Meaning:**

These findings suggest that disadvantaged areas are likely to experience shortages in dental care and disparities in spatial access to dental services.

## Teaser

This cross-sectional study evaluates the spatial accessibility of US dental clinics and the characteristics of counties and block groups with dental care shortage areas.

## Introduction

Considerable efforts have been dedicated to addressing disparities in oral health for low-income and disadvantaged racial and ethnic minority populations.^1,2^ Less attention has been devoted to addressing geographic barriers and the availability of dental clinicians in underserved areas. These barriers can impact the ability of individuals in these regions, particularly vulnerable populations such as older adults and those with low income, to access dental care, leading to delays in seeking care and poor oral health outcomes.^3^

Spatial accessibility to health care refers to the availability of clinics and practitioners as well as the accessibility and travel distance or time to health care facilities.^4,5,6^ Many of the current methods for measuring accessibility have significant drawbacks. For example, the clinician-to-population ratio fails to account for patients who travel across administrative geographic boundaries to access health care. Similarly, measuring the distance to the nearest clinic ignores that patients can bypass the nearest clinic in favor of their preferred one. Calculating the mean distance or travel time disregards patients’ tendency to cross geopolitical boundaries such as counties. Frequently used distance and travel time-based approaches also fail to take into account demand (number of people needing dental care) and supply (number of dentists) factors.^4^

The Health Resources and Services Administration (HRSA) designates counties as Health Professional Shortage Areas (HPSAs) for dental services, using clinician-to-population ratio and travel time to the nearest clinic.^7^ According to HRSA’s most recent data, there are 6854 HPSAs in 2024 with 58 million people in the US living in these areas.^8,9^ However, HRSA designations disregard the relationships between the demand and supply of clinicians and the cross-county boundary access. HRSA’s approach also considers the distance to the nearest clinic within a county without considering the availability of multiple clinics within a convenient distance. These shortcomings can lead to unrealistic dental market boundaries,^10^ coarse aggregations of data on shortages,^7^ and a lack of correlation with actual dental care use.^11,12^

In this study, we used an advanced gravity-based approach, the enhanced 2-step floating catchment area (E2SFCA) that considers the availability of clinicians (both location and the size of dental clinics), accessibility (travel time or distance), and adjustments for demand and supply factors through distance-decay weights.^4,13,14^ We examined the spatial accessibility to dental care nationally in the US at the block group level, using a national dataset with the precise location of dentists and spatial accessibility within a 30-minute drive.^14,15^ Census blocks are the smallest geographic units used by the US Census Bureau, while block groups are aggregations of these blocks typically containing between 600 and 3000 people.^16^ Our focus on spatial accessibility at the block group level offers a more detailed and precise analysis compared with previous studies at census tract and ZIP code levels.^17,18^ We also examined the characteristics of counties and block groups with dental care shortage areas. Evaluating spatial accessibility and shortage areas can inform policy efforts for better resource allocation and an effective health care system that can meet the needs of all populations.

## Methods

This cross-sectional study was determined to be not human participants research and therefore did not require informed consent by the institutional review board of the Harvard Faculty of Medicine. We followed the Strengthening the Reporting of Observational Studies in Epidemiology (STROBE) reporting guidelines.^19^

### Data Sources

#### Dentists Database

We used the IQVIA national dentists’ database, which includes dentists from all 50 states and Washington, DC. This database includes detailed information on dentists, such as specialties, license status, and practice location. The IQVIA database is a comprehensive database of all active dentists, including safety-net clinics, federally qualified health centers, and university-based clinics, which was created from over 1000 government and industry sources.^20^ The database is continuously updated and validated through medical and prescription claims, verification with state boards, telephone calls, and web research.^21,22,23^ We used the IQVIA database that was last updated in October 2023.

We excluded 10 275 inactive dentists, such as retired dentists. Our database included 205 762 dentists, including 196 756 general dentists and 9006 specialists. Using available coordinates and ArcGIS World Geocoding Services,^24^ we converted the dentists’ addresses to their absolute locations.

#### Socioeconomic Data

We linked IQVIA data to the US Census Bureau’s Topologically Integrated Geographic Encoding and Referencing (TIGER/Line Shapefiles), which includes information on geographic boundaries for various US Census levels.^25^ For each block group and county, we obtained data on the racial and ethnic composition, population by age group, poverty level, educational attainment, median household income, and health insurance from the 5-year American Community Survey (ACS) 2022 estimates.^26,27,28,29,30,31^ Demographic and housing characteristics data at the block level were obtained from the decennial census in 2020 to calculate the block group population mean center.^32^ In addition, urban and rural population data based on the 2020 census were obtained from the Environmental Systems Research Institute (ESRI)’s online data portal.^33^

We also used the Area Deprivation Index (ADI) version 3.1, which measures neighborhood socioeconomic disadvantage at the block group level using 17 indicators.^34,35^ The ADI index ranks block groups from 1% (least disadvantaged) to 100% (most disadvantaged).^35^

#### Racial and Ethnic Segregation

We used the dissimilarity index, a measure of evenness, to measure the residential segregation of Hispanic and non-Hispanic Black populations from the White population. The dissimilarity index estimates the proportion of each race, such as non-Hispanic Black, that would need to relocate to achieve a more even spatial distribution within a specific geographic unit. We calculated the dissimilarity index at both the block group and county levels. The index ranges from 0 to 1, with 0 indicating complete integration and 1 indicating complete segregation.^36^

#### Accessibility Measurement

We used an E2SFCA gravity model to calculate spatial accessibility scores for each population center. The catchment area was defined by the 30-minute drive time similar to previous studies,^14,15^ which includes all areas reachable within a 30-minute drive from a dental clinic service catchment area (details in eMethods in Supplement 1). Using the origin-destination cost matrix of ArcGIS Pro software,^37^ we calculated the drive time between population centers and dental clinics. For consistency, we calculated all driving distances and drive times at 10:00 am on a Tuesday to avoid rush hour traffic in major cities. We used the Street Map Premium Network 2023 version as the underlying network dataset for travel time calculations.^38^

We categorized the accessibility scores into 4 groups: (1) dental desert: block groups with an accessibility score of 0, indicating that the population in that block group had no clinics within a 30-minute drive; (2) limited access: block groups with accessibility scores between 0 and 0.0002. This threshold is based on HPSA designation^8^; (3) adequate access: block groups with an accessibility score of between 0.0002 and less than twice the minimum clinician-to-population ratio (0.0004); (4) extensive access: block groups with an accessibility score of over 0.0004. This categorization of accessibility scores is informed by both data-driven insights and literature on health care accessibility. Specifically, the threshold of 0.0002 is aligned with HPSA criteria reflecting standards in clinician-to-population ratios.^8,39^

#### Inequality in Access to Dental Care Measurement

We measured the inequality in access to dental clinics at both the state and county levels using the Gini coefficient of the accessibility score.^40,41^ We calculated the Gini coefficient using accessibility scores at the block group level derived from E2SFCA. To account for the spatial contiguity of adjacent census block groups, we used a queen’s case contiguity weight to calculate a spatially adjusted Gini coefficient.^42^

### Statistical Analyses

We examined accessibility to dental clinics including all dentists (general and specialists). In a secondary analysis, we limited the population to include only general dentists and the adult population to measure adults’ access to routine dental care. We did not examine the accessibility to pediatric dental clinics because many general dentists accept children, and we could not identify these dentists in our data.

We examined 3 outcomes: dental care shortages at the block group level and at the county level, and inequality in accessibility to dental clinics at the county level. We defined dental care shortages at the block group level by combining dental deserts and areas with limited access. Dental care shortages at the county level were counties with more than 50% of their population living in shortage block groups. We identified counties with unequal access to dental clinics if the Gini accessibility coefficient exceeded 0.4, as this threshold indicates a transition beyond adequate equality.^43^

We defined rural areas as areas where more than half of the population was rural in a geographic unit.^33^ We calculated the percentage of the uninsured population and normalized the percentage using the *z* score method. Black and Hispanic segregation was defined as the fourth quartile of the dissimilarity index for each group, with nonsegregation (first to third quartiles) as our reference group.^44^ We calculated ADI at the county level by taking each county’s population-weighted mean of block-group ADI. We sorted block groups and counties according to ADI quartiles and used the first quartile (least disadvantaged counties) as our reference group.

Finally, we estimated linear regression models to examine the characteristics of block groups and counties associated with dental care shortage areas and inequality in access. The regression model at the block group level included block group–level covariates of rurality, population density, percentage of uninsured population, Hispanic and Black segregation (dissimilarity index), and the ADI. The regression model at the county level used the same covariates but at the county level. All analyses used robust SEs clustered by the census for the block group–level model and by states for the county-level models.^45^ Statistical significance was based on a 2-sided *P* ≤ .05.

We merged demographic and socioeconomic data using geographic identifiers at the block group and county level. We used Python version 3.12.1^46^ (Python Software) and ArcGIS Pro version 3.2.2^47^ (Esri) for all analyses. Data were analyzed from November 2023 to April 2024.

## Results

### Spatial Accessibility at the Block Group Level

**Figure 1.**
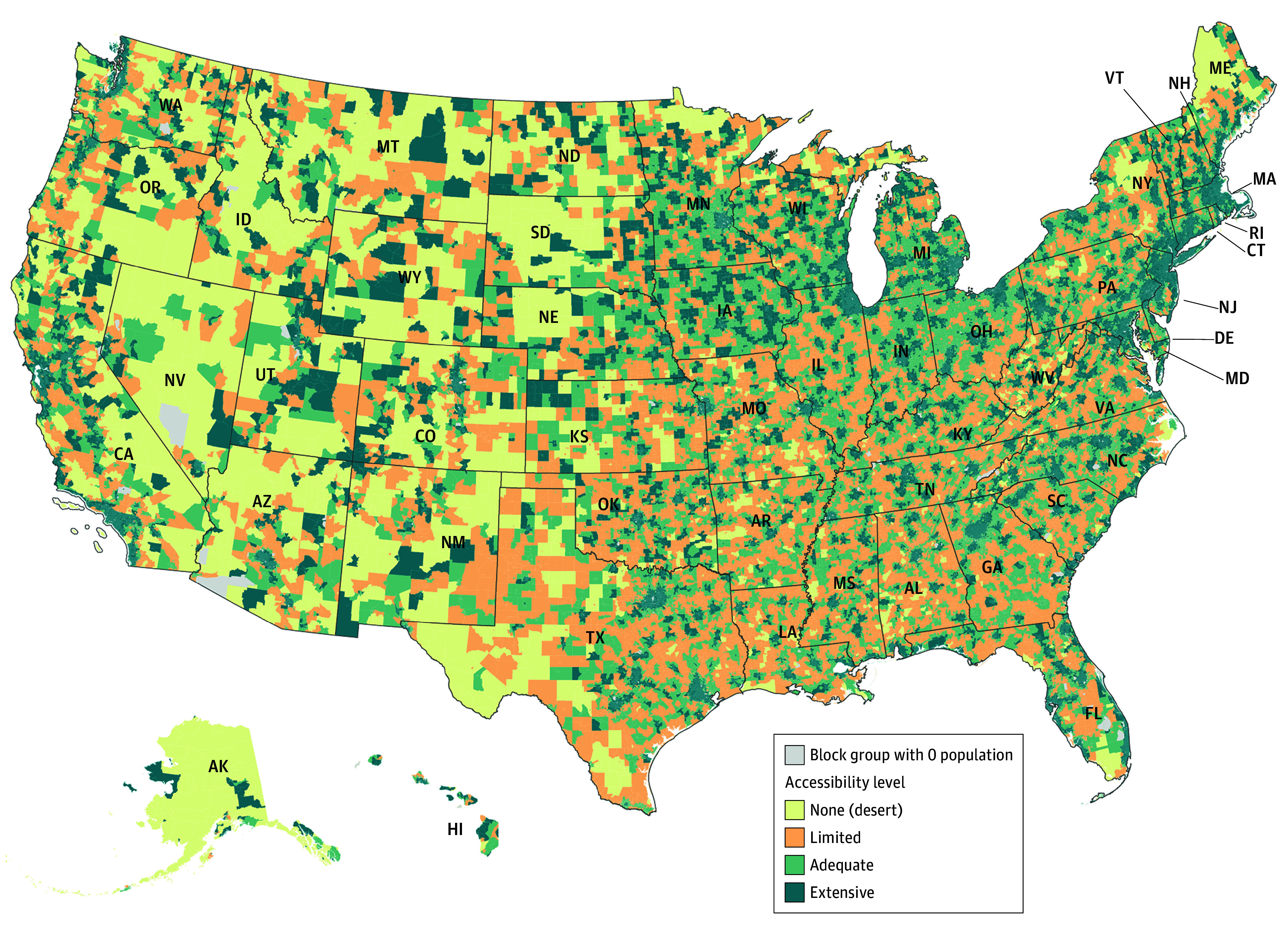
Dental Deserts and Dental Care Shortage Areas at the Block Group Level Accessibility analysis considering all dentists (general and specialty clinics). Authors’ analysis of data from (1) dentist database from IQVIA; (2) American Community Survey 2022 estimates of population and Topologically Integrated Geographic Encoding and Referencing/line shapefiles from US Census Bureau. Categories are based on the following accessibility scores: 0 = no access; greater than 0 to less than 0.0002 = limited access; greater than or equal to 0.0002 to less than 0.0004 = adequate access; and greater than or equal to 0.0004 = extensive access.

Figure 1 illustrates the variation in levels of spatial accessibility at the block group level. We found that 4 states (Connecticut, Delaware, Indiana, and New Jersey) and Washington, DC did not have dental deserts. On the other hand, Alaska had the highest percentage of the population (76 187 people [10.4%]) living in dental desert areas, followed by Montana (85 566 people [7.8%]) and North Dakota (59 988 people [7.7%]) (eFigure 1 in Supplement 1).

Nearly 1.7 million people (0.5%) did not have access to dental clinics within a 30-minute drive. Additionally, 24.7 million (7.5%) lived in dental care shortage areas and 387 counties (12.3%) had significant disparities in access to dental clinics. On the other hand, over 56.4 million people (17.0%) had adequate access to dental clinics, and over two-thirds (249.9 million people [75.5%]) had extensive access to dental clinics.

Most block groups (306.3 million [92.5%]) with extensive access to dental clinics were in urban areas. We found a significant difference in spatial accessibility scores between rural and urban areas, with 1 dentist for every 3850 people in rural areas compared with 1 dentist for every for 1470 people in urban areas (eFigure 2 in Supplement 1). For adults’ accessibility (eFigure 3 in Supplement 1), we found that 193.4 million adults (94.9%) had adequate access to dental clinics, while 9.7 million adults (5.0%) lived in dental care shortage areas.

### Spatial Accessibility at the County Level

**Figure 2.**
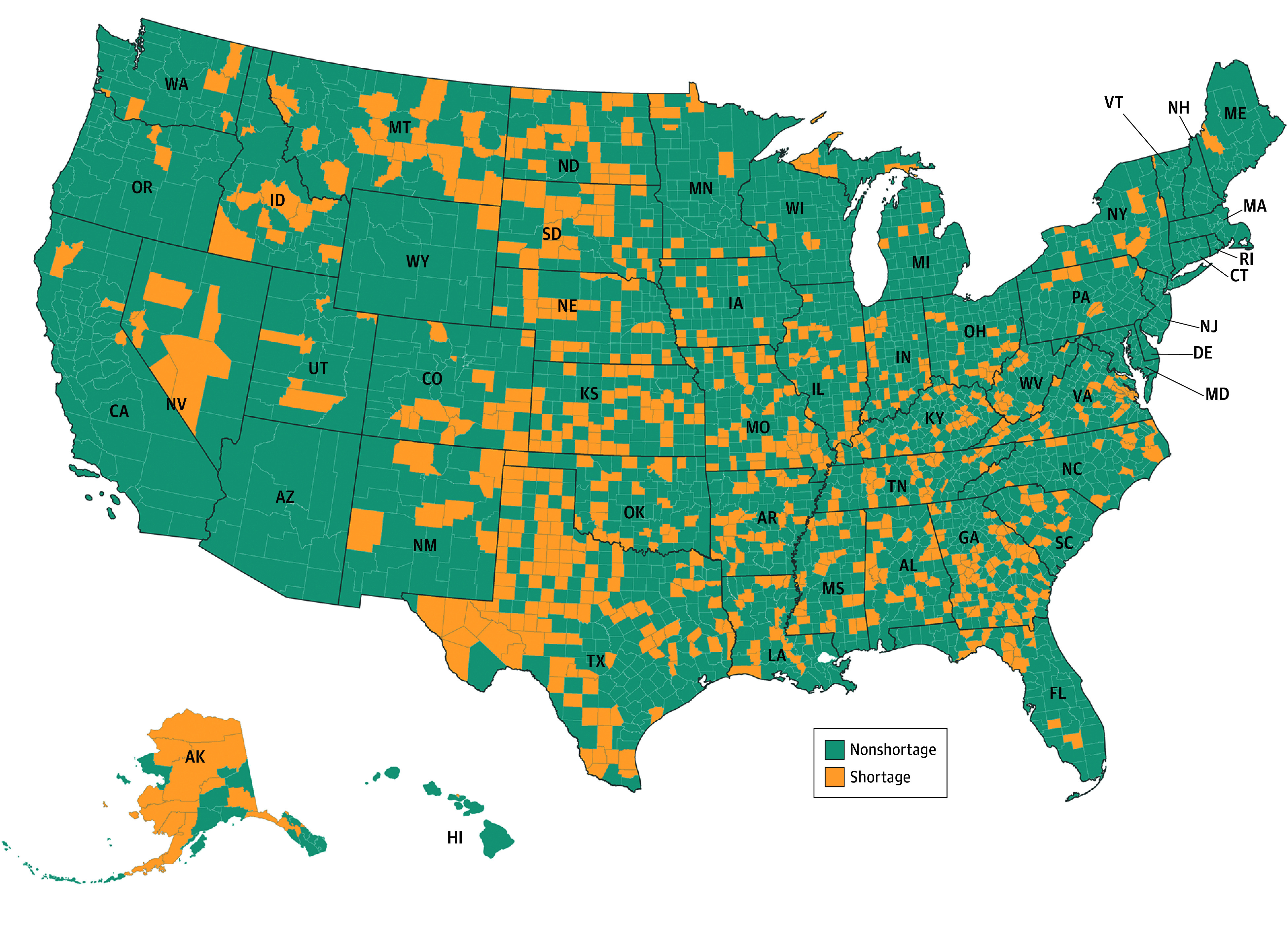
County-Level Dental Care Shortage Areas Accessibility analysis considering all dentists (general and specialty clinics). Authors’ analysis of data from (1) dentist database from IQVIA; (2) American Community Survey 2022 estimates of population and Topologically Integrated Geographic Encoding and Referencing/line shapefiles from US Census Bureau. Shortage counties are counties with more than 50% of the population living in shortage areas.

We found that 787 of the 3143 counties had a shortage of dentists (Figure 2), with nearly 3.2% of the population (10.6 million) living in these counties. Although Black individuals account for nearly 12% of the US population, only 1.1 million (0.3%) resided in shortage counties. Similarly, despite Hispanic individuals comprising approximately 19% of the overall US population, they represented just 0.9 million (0.3%) of the residents in shortage counties. These shortage counties had a low population density, with only 27 people per square kilometer compared with 359 people in nonshortage areas. Furthermore, nearly 15.6% of the population in shortage counties lived below the federal poverty level. Approximately 9.8 million people (92.2%) resided in rural counties experiencing a shortage of dental care (Table 1).

**Table 1.** Socioeconomic Characteristics of Dental Care Shortage Areas

Characteristic	Individuals, No. in millions (%)					
	Block group level^a^		County-level^b^		County-level inequality of accessibility^c^	
	Shortage	Nonshortage	Shortage	Nonshortage	Inequality	Equality
Population, millions	24.7 (7.5)	306.3 (92.5)	10.6 (3.2)	320.4 (96.8)	3.9 (1.2)	327.1 (98.8)
Race and ethnicity						
Non-Hispanic White	18.5 (74.9)	173.1 (56.5)	8.0 (75.5)	183.7 (57.3)	2.8 (72.6)	188.9 (57.7)
Non-Hispanic Black	1.7 (6.9)	38.2 (12.48)	1.1 (9.9)	38.9 (12.1)	0.2 (5.3)	39.7 (12.1)
Hispanic	2.8 (11.5)	59.2 (19.3)	0.9 (8.7)	61.1 (19.1)	0.3 (7.8)	61.7 (18.9)
Population density, mean (SD)^d^	143 (640)	2719 (6317)	27 (28)	359 (2125)	12 (18)	313 (1968)
Percentage below the federal poverty level, mean (SD)	14.3 (12.1)	13.1 (13.8)	15.6 (6.8)	13.8 (5.7)	15.1 (7.5)	14.2 (5.8)
Median household income, mean (SD), US $^e^	66 267 (26 158)	84 346 (42 762)	56 981 (13 566)	65 409 (17 218)	57 957 (14 397)	64 061 (16 950)
% Education level, mean (SD)^f^	13.2 (10.8)	11.1 (11.7)	13.7 (6.8)	11.0 (5.1)	11.7 (6.4)	11.6 (5.6)
Rural, millions	20.8 (83.9)	42.8 (14.0)	9.8 (92.2)	37.2 (11.6)	3.6 (93.1)	43.4 (13.3)
Uninsured population, millions	2.5 (10.1)	25.8 (8.4)	1.1 (10.5)	27.2 (8.5)	0.4 (10.5)	27.9 (8.5)
Public health insurance, millions^g^	5.7 (22.9)	61.9 (20.2)	2.5 (23.6)	65.0 (20.3)	1.0 (25.9)	66.5 (20.3)

^a^ Dental care shortage areas are block groups with accessibility scores less than 0.0002.

^b^ Counties where more than 50% of the population were living in dental care shortage block groups.

^c^ Counties with Gini coefficient of accessibility scores greater than 0.4 have inequality in access.

^d^ The number of people per square kilometer of land area.

^e^ The mean of median household income in US dollars.

^f^ The percentage of the population with below high school education.

^g^ Public health insurance includes Medicaid and Medicare.

The percentage of population without health insurance was higher in shortage counties (1.1 million [10.5%]) than in nonshortage counties (27.2 million [8.5%]). Around 2.5 million people (23.6%) with Medicaid or Medicare health insurance resided in shortage counties.

### Inequality in Access to Dental Clinics

**Figure 3.**
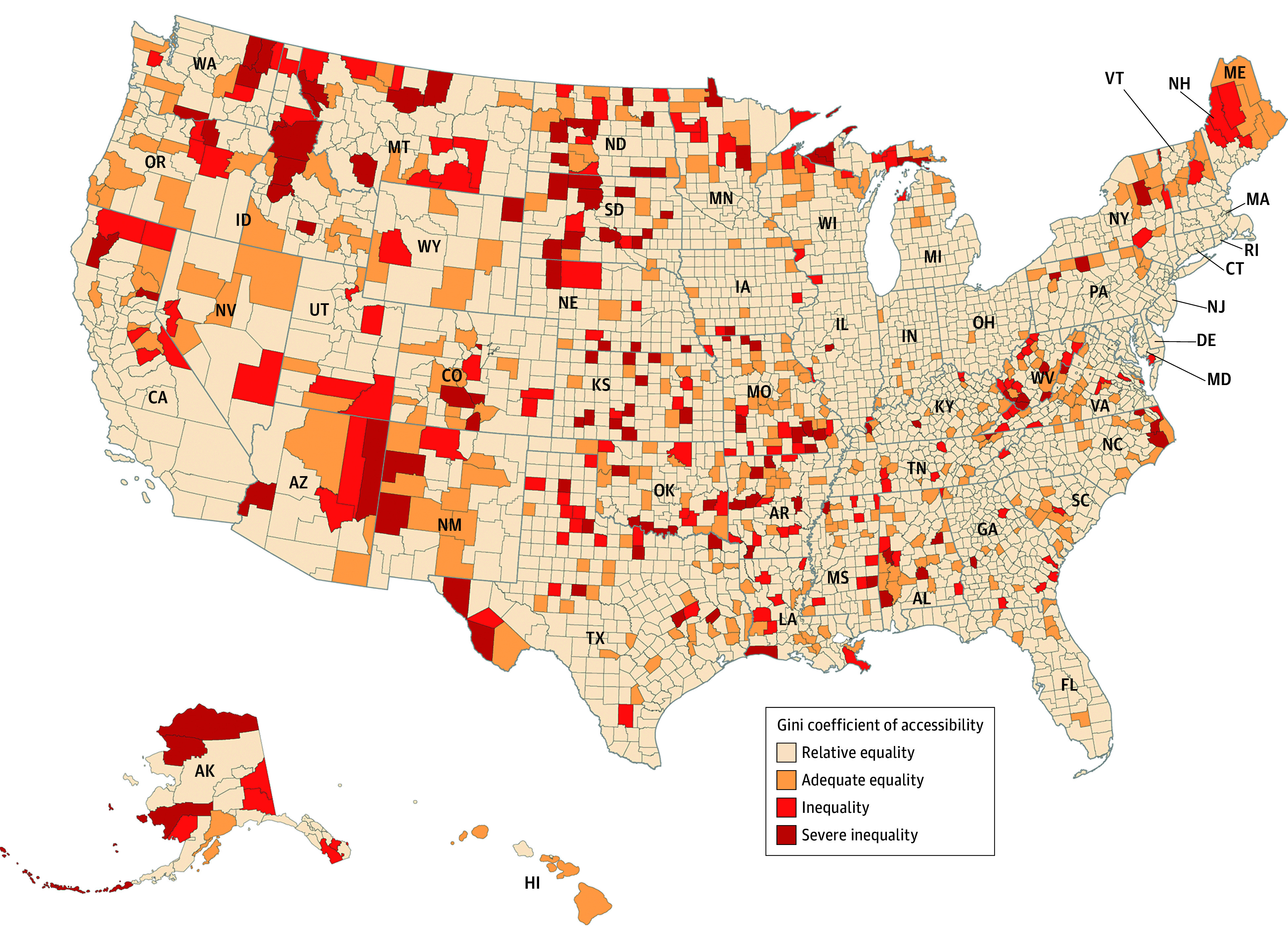
County-Level Gini Coefficients of Accessibility Scores Accessibility analysis considering all dentists (general clinics and specialty clinics). Authors’ analysis of data from (1) dentist database from IQVIA; (2) American Community Survey 2022 estimates of population and Topologically Integrated Geographic Encoding and Referencing/line shapefiles from US Census Bureau. The Gini coefficient at the county level is calculated using accessibility scores at the block group level.

There were 387 counties out of 3143 with a Gini accessibility coefficient greater than 0.4, indicating inequality in access to dental clinics (Figure 3). Approximately 3.9 million (1.2%) of the US population resided in these counties with uneven spatial accessibility. These areas had low population density and were mostly rural. eFigure 4 in Supplement 1 highlights that Washington, DC, stood out with its evenly distributed accessibility, as 1 dentist was available for less than 1000 people. On the other hand, Arkansas, Alabama, Mississippi, and West Virginia showed high Gini values and low accessibility, indicating an unequal distribution of dental clinics.

### Characteristics of Areas With Dental Care Shortages and Inequalities in Access to Dental Clinics

Our regression analyses (Table 2) showed that rural block groups were 23.9 percentage points (pp) (95% CI, 23.6 to 24.3 pp) more likely to be dental care shortage areas compared with urban block groups. Block groups with high levels of Black and Hispanic segregation were 1.5 pp (95% CI, 1.3 to 1.7 pp) and 4.5 pp (95% CI, 4.3 to 4.8 pp) more likely to have dental care shortages, respectively, compared with less segregated block groups. Block groups with the highest level of socioeconomic deprivation (fourth ADI quartile) were 5.5 pp (95% CI, 5.1 to 5.9 pp) more likely to experience dental care shortages than the least deprived block groups. In contrast, densely populated areas were less likely to have dental care shortage areas (−0.4 pp; 95% CI, −0.6 to −0.3 pp) than low-density areas.

**Table 2.** Characteristics of Areas With Dental Care Shortages and Inequalities in Access to Dental Care^a^

Characteristic	Dental care shortage areas				Inequality in access	
	Block group level (n = 21 290 block groups)^b^		County level (n = 787 counties)^c^		County level (n = 387 counties)^d^	
	Coefficient (95% CI)	*P* value	Coefficient (95% CI)	*P* value	Coefficient (95% CI)	*P* value
Rural	0.24 (0.24 to 0.24)	<.001	0.23 (0.19 to 0.26)	<.001	0.11 (0.09 to 0.14)	<.001
Population density	−0.00 (−0.01 to −0.00)	<.001	−0.00 (−0.05 to 0.04)	.95	−0.00 (−0.04 to 0.03)	.85
Uninsured population, %	0.00 (0.00 to 0.00)	<.001	0.03 (0.01 to 0.05)	.002	0.03 (0.02 to 0.04)	<.001
Black segregation						
Dissimilarity index (1st-3rd quartiles)	1 [Reference]		1 [Reference]		1 [Reference]	
Dissimilarity index (4th quartile)	0.02 (0.01 to 0.02)	<.001	−0.02 (−0.06 to 0.01)	.15	0.01 (−0.02 to 0.03)	.48
Hispanic segregation						
Dissimilarity index (1st-3rd quartile)	1 [Reference]		1 [Reference]		1 [Reference]	
Dissimilarity index (4th quartile)	0.04 (0.04 to 0.05)	<.001	−0.08 (−0.11 to −0.05)	<.001	−0.02 (−0.04 to 0.01)	.13
Area deprivation index						
1 (Least deprived)	1 [Reference]		1 [Reference]		1 [Reference]	
2	0.01 (0.00 to 0.01)	<.001	0.02 (−0.02 to 0.64)	.33	0.01 (−0.02 to 0.04)	.69
3	0.03 (0.25 to 0.03)	<.001	0.09 (0.04 to 0.13)	<.001	0.01 (−0.02 to 05)	.50
4 (Most deprived)	0.06 (0.05 to 0.06)	<.001	0.20 (0.15 to 0.25)	<.001	0.06 (0.02 to 0.09)	.002

^a^ Accessibility analysis considering all dentists (general clinics and specialty clinics). Authors’ analysis of data from (1) dentist database from IQVIA; (2) American Community Survey 2022 estimates of population and the Topologically Integrated Geographic Encoding and Referencing/line shapefiles from the US Census Bureau. The Gini coefficient at the county level is calculated using accessibility scores at the block group level.

^b^ Dentist shortage areas are block groups with accessibility scores less than 0.0002.

^c^ Dentist shortage areas are counties with more than 50% of the county’s population living in dentist shortage block groups.

^d^ Inequality in accessibility are counties with a Gini coefficient of accessibility scores greater than 0.4.

We estimated that there were 2818 counties where at least 10.0% of people lived in dental care shortage block groups. At the county level, counties with a dental care shortage were more likely to be rural (22.6 pp; 95% CI, 19.3 to 26.0 pp), have an uninsured population (3.0 pp; 95% CI, 1.1 to 4.9 pp), and be socioeconomically deprived (20.0 pp; 95%CI, 15.2 to 24.8 pp). However, counties with Hispanic segregation areas were less likely (−8.0 pp; 95% CI, −11.4 to −4.5 pp) to have dental care shortages. Rural counties (11.3 pp; 95% CI, 8.9 to 13.7 pp), counties with high uninsured populations (3.0 pp; 95% CI, 1.5 to 4.4 pp), and socioeconomically deprived counties (5.8 pp; 95% CI, 2.1 to 9.5 pp) were more likely to have inequalities in access to dental clinics.

## Discussion

In this cross-sectional study, we found around 24.7 (7.5%) million people in the US lived in areas with a shortage of dental services and 387 (12.3%) counties had significant disparities in access to dental clinics. Counties that were rural, deprived, and had a high proportion of uninsured people were more likely to experience a shortage of dentists and spatial disparities in access. Our findings also highlight the intersection of rurality and race and ethnicity. We found that more White populations lived in counties with a shortage of dentists, as compared with Hispanic and Black populations. This is mainly because rural areas, which often face such shortages, tend to have a higher concentration of White residents. However, in urban areas where there is segregation and a concentration of poverty, Hispanic and Black individuals were more likely to live in areas with a shortage of dental care.^48,49^

Our estimates of dental care shortage areas are higher than those estimated by HRSA geographic shortage areas^39^ but similar to those of other state-focused studies in Missouri and Wisconsin.^15^ However, our estimate that there were 2818 counties where at least 10.0% of people lived in dental care shortage block groups is close to the number of counties (2412) that HRSA identified as population shortage HPSAs.^50^ When comparing our results with HRSA’s HPSA definition, it is important to note the differences in definitions and methodologies. Our study uses the E2SFCA method, which focuses on spatial accessibility at the block group level. This approach considers multiple detailed factors such as clinician availability, travel time, distance decay, and cross-county movement, resulting in a more precise and comprehensive assessment of dentist availability. In contrast, HRSA dental care shortage areas use broader criteria based on county-level data.

We found nearly 12.3% of counties had unequal access to dental care, with dental clinics concentrated in a few areas, highlighting systemic issues with the distribution of the dental care workforce. As expected, these counties were mostly rural and had low population density, which further supports the long-standing concern that rural areas are deprived of adequate access to dental care. Addressing this geographic maldistribution requires comprehensive workforce planning to create a supportive work environment to recruit and retain dentists.^51^ This could include increasing funding for dental education, offering more incentives for practitioners to work in underserved areas such as enhanced funding to the National Health Service Corps, expanding training in current dental care shortage areas, and expanding the breadth and scope of the dental workforce to include dental therapists or advanced dental hygienists. Moreover, strategies such as telemedicine, community health programs, and mobile clinics can help ensure equitable access to dental services in rural and underserved areas.^52^

Findings from our primary analysis examining the spatial accessibility of dental clinics for all populations and dentists were similar to our secondary analyses, which focused only on the adult population and general dentists, excluding specialty-only clinics. These findings suggest that the underlying factors affecting the availability and distribution of dentists are pervasive across specialties. These geographic barriers can lead to longer wait times, reduced quality of care, and persistent disparities in oral health, as patients may struggle to find timely and appropriate dental care.

Our analyses at the block group level indicated that dental care shortage areas were more likely to be segregated areas with a high segregation of Black and Hispanic populations. However, this pattern was not observed at the county level, emphasizing the importance of examining smaller geographic units. We also found, as expected, that dental care shortage areas were mostly rural. Future studies examining racial and ethnic disparities in spatial access to dental care can focus on metropolitan areas with large racial and ethnic minority populations to understand the precise demand of the population at a more detailed level, such as a 100-m grid, rather than just at the block group level.

We used travel time to define our catchment area and calculate the accessibility score over drive distance because our research focuses on rural and urban areas. However, there is no standard recommendation for determining an optimum travel time to dental clinics. Previous studies have used various thresholds to measure accessibility to health care clinics.^14,15,18,53,54,55,56,57^ Studies have shown that, on average, residents of the US spend 22.0 to 27.1 minutes on travel for medical and dental care.^58,59,60^ Moreover, the US Department of Veterans Affairs defined a 30-minute drive time as the standard for appropriate access.^61^ Therefore, we used 30 minutes as the travel time threshold for our analyses.

### Limitations

This study had limitations. Calculation of drive-time is a standard and widely accepted approach in spatial accessibility research allowing for consistent comparison with other studies.^13,14,15,18^ However, a key limitation is that it assumes all individuals have access to a car and the calculations do not account for transportation barriers faced by individuals with low-incomes, older adults, and children who may lack such access, especially in rural areas. Our analyses assume that all populations reside at the center of the block group. Additionally, we considered all practitioners to be working full-time, which may have underestimated dental care shortage areas in our analysis. Furthermore, our analyses only focus on spatial accessibility to dental clinics without considering other factors such as dental insurance coverage, transportation, and health literacy, which also affect accessibility. Additionally, our regression models only examine the association between our outcomes and neighborhood socioeconomic factors and cannot determine any causal effects.

## Conclusions

Little is known about spatial accessibility to dental clinics within a convenient travel time nationally for the US. This cross-sectional study of US dental clinics is the first we know of to use the precise location of dental clinics to map spatial accessibility at the block group level nationally. We found variations in dentist distribution across counties and block groups, indicating a geographic maldistribution of the dental workforce. We also found shortage areas were likely to be rural, deprived, and have a high proportion of uninsured people and were more likely to experience spatial disparities in access to dental care. Our findings can support dental workforce planning efforts and targeted interventions at the federal and state levels to encourage dentists to practice in underserved areas to reduce disparities in access to dental care.

## Notes

Supplement 1.
eMethods.

eReferences.
**eFigure 1.** The Percentage of the Population According to Accessibility Level to Dental Care in 50 States and Washington, DC **eFigure 2.** The Distribution of Accessibility Scores to Dental Care in Rural and Urban Areas **eFigure 3.** Dental Deserts and Dental Care Shortage Areas at the Block Group Level **eFigure 4.** Mean Clinician-to-Population Ratio According to the Gini Coefficient of Accessibility

Supplement 2.
Data Sharing Statement
