# Supplementary material for: Dental Clinic Deserts in the US: Spatial Accessibility Analysis
Source: JAMA Netw Open. 2024 Dec 23;7(12):e2451625. doi: 10.1001/jamanetworkopen.2024.51625 (PMC11667347; doi:10.1001/jamanetworkopen.2024.51625)
Supplement: Supplement 1. — eMethods. eReferences. eFigure 1. The Percentage of the Population According to Accessibility Level to Dental Care in 50 States and Washington, DC eFigure 2. The Distribution of Accessibility Scores to Dental Care in Rural and Urban Areas eFigure 3. Dental Deserts and Dental Care Shortage Areas at the Block Group Level eFigure 4. Mean Clinician-to-Population Ratio According to the Gini Coefficient of Accessibility [file jamanetwopen-e2451625-s001.pdf]

Supplemental Online Content

Rahman MS, Blossom JC, Kawachi I, Tipirneni R, Elani HW. Dental clinic deserts in the US: spatial accessibility analysis. *JAMA Netw Open*. 2024;7(12):e2451625. doi:10.1001/jamanetworkopen.2024.51625

eMethods.
eReferences.
eFigure 1. The Percentage of the Population According to Accessibility Level to Dental Care in 50 States and Washington, DC
eFigure 2. The Distribution of Accessibility Scores to Dental Care in Rural and Urban Areas
eFigure 3. Dental Deserts and Dental Care Shortage Areas at the Block Group Level
eFigure 4. Mean Provider-to-Population Ratio According to the Gini Coefficient of Accessibility

This supplemental material has been provided by the authors to give readers additional information about their work.

© 2024 Rahman MS et al. *JAMA Network Open.*

eMethods.

Selection of 30-minute Travel Impedance

The outcome of a gravity-based model like an Enhanced Two-step Floating Catchment Area (E2SFCA) is sensitive to travel impedance. Therefore, it is important to select the appropriate threshold of accessibility impedance (travel time/distance). Patients can travel more distances in rural areas than in urban areas within the same driving time because of traffic congestion in urban areas. Therefore, we used travel time instead of drive distance as the travel impedance since our research focuses on both rural and urban areas in the US. Previous studies have used thresholds of 5, 10, 15, 20, 30, and 60 minutes to measure accessibility to healthcare facilities in the US and other countries, depending on the study's objectives, the types of service, and the geographic characteristics of study areas.[1–8] Although there is no consensus on travel time duration as the optimum threshold for healthcare accessibility, 30-minutes is frequently used as a threshold. A 2017 national household travel survey found that, on average residents of the US spent 27.1 minutes (urban 25.5 and rural 34.2) to access medical and dental care.[9] Another study reported 22 minutes as the nationwide average for trips to medical and dental care (urban 20.7 minutes and urban 27.2 minutes).[10] In Washington, residents are willing to travel an average of 28.4 minutes for routine care[11]. Many studies defined 30 minutes as the cutoff for the travel burden for medical and dental care, and it is estimated that 7.9 % of the US population needs to travel more than 30 minutes.[9,10] Although there is no universally accepted recommendation for the optimum travel time to access dental care, we used a 30-minute travel time impedance threshold based on available literature and empirical evidence.

Mapping Dental Clinics

To determine the spatial accessibility of dental clinics, we used a 30-minute drive time to dental clinics. We aggregated dental practitioners within each dental service-providing clinic. We used the unique Street Front ID of dentists from the IQVIA dataset to aggregate dentists at the clinic level or the suitability of the travel time calculation between the population center and clinics. To determine the supply of dentists at the clinic level, we considered the total number of dentists and the number of dentists in each specialty group for each clinic location.

Mapping Population Center

We assumed that all individuals residing within a block group are at the population-weighted mean center of the corresponding block group. We refer to this center as the population center for simplicity. To calculate the geographic coordinates of the population-weighted mean center for each block group, we used the population-weighted sum of latitudes and longitudes of the geometric center of all census blocks within the corresponding block group. It is important to note that if all blocks within a block group have zero population, the geometric center of the block group is treated as the population mean center since the population weight is zero in this specific case.

Detailed explanation of the E2SFCA method

© 2024 Rahman MS et al. *JAMA Network Open.*

In the first step of E2SFCA, we calculated the ratio between the supply of providers and the demand for a clinic's population using Equation 1.

© 2024 Rahman MS et al. *JAMA Network Open.*

$$R_j = \frac{S_j}{\sum_{i=1}^{n} W_{ij} P_i} \qquad \textit{Equation 1}$$

Where R_j is the supply-demand ratio for the clinic j, P_i is the number of people living in population center i, W_{ij} is the Gaussian distance decay weight between the clinic j and the population center i, and n is the number of population center within the service area catchment of the clinic j. The Gaussian distance decay weight was calculated using the following equation 2; where t_{ij} is the drive time in minutes between population center i and the clinic j, and t_0 is the 30-minute threshold.

$$W_{ij} = \begin{cases} \dfrac{e^{-\frac{1}{2}\times\left(\frac{t_{ij}}{t_0}\right)^2} - e^{-\frac{1}{2}}}{1 - e^{-\frac{1}{2}}}, & t_{ij} < t_0 \\ 0, & t_{ij \geq t_0} \end{cases} \qquad \textit{Equation 2}$$

In second step, the accessibility scores are calculated at the census block group level by taking the weighted sum of the supply-demand ratios of all clinics within the 30-minute catchment area of the corresponding block group (Equation 3).

$$AS_i = \sum_{j=1}^{m} W_{ij} \times R_j \qquad \textit{Equation 3}$$

Where, AS_i is the accessibility score at block group i and m is the number of clinics within 30 minutes' drive time from the population center.

eReferences

1. Luo W, Qi Y. An enhanced two-step floating catchment area (E2SFCA) method for measuring spatial accessibility to primary care physicians. *Health & place*. 2009;15(4):1100-1107. doi:10.1016/j.healthplace.2009.06.002

2. Nasseh K, Eisenberg Y, Vujicic M. Geographic access to dental care varies in Missouri and Wisconsin. *J Public Health Dent*. 2017;77(3):197-206. doi:10.1111/jphd.12197

3. Wang F, Luo W. Assessing spatial and nonspatial factors for healthcare access: towards an integrated approach to defining health professional shortage areas. *Health & place*. 2005;11(2):131-146. doi:10.1016/j.healthplace.2004.02.003

4. Kiani B, Mohammadi A, Bergquist R, Bagheri N. Different configurations of the two-step floating catchment area method for measuring the spatial accessibility to hospitals for people living with disability: a cross-sectional study. *Arch Public Health*. 2021;79(1):85. doi:10.1186/s13690-021-00601-8

5. Yerramilli S, Fonseca DG. Assessing geographical inaccessibility to health care: Using GIS network based methods. *Public Health Research*. 2014;4(5):145-159. doi:10.5923/j.phr.20140405.01

6. McGrail MR, Humphreys JS. Measuring spatial accessibility to primary care in rural areas: Improving the effectiveness of the two-step floating catchment area method. *Applied Geography*. 2009;29(4):533-541. doi:10.1016/j.apgeog.2008.12.003

7. Bauer J, Groneberg DA. Measuring spatial accessibility of health care providers–introduction of a variable distance decay function within the floating catchment area (FCA) method. *PloS one*. 2016;11(7):e0159148. doi:10.1371/journal.pone.0159148

© 2024 Rahman MS et al. *JAMA Network Open.*

8. Naylor KB, Tootoo J, Yakusheva O, Shipman SA, Bynum JP, Davis MA. Geographic variation in spatial accessibility of US healthcare providers. *Plos one*. 2019;14(4):e0215016. doi:10.1371/journal.pone.0215016

9. Akinlotan M, Khodakarami N, Primm K, Bolin J, Ferdinand AO. Travel for medical or dental care by race/ethnicity and rurality in the US: Findings from the 2001, 2009 and 2017 National Household Travel Surveys. *Preventive Medicine Reports*. 2023;35:102297. doi:10.1016/j.pmedr.2023.102297

10. Probst JC, Laditka SB, Wang JongYi WJ, Johnson AO. *Mode of Travel and Actual Distance Traveled for Medical or Dental Care by Rural and Urban Residents.* South Carolina Rural Health Research Center; 2006. Accessed July 5, 2024. https://www.sc.edu/study/colleges_schools/public_health/research/research_centers/sc_rural_health_research_center/documents/61modeoftravelandactualdistancetraveled2006.pdf

11. Yen W. How long and how far do adults travel and will adults travel for primary care? Published online 2023. Accessed April 17, 2024. https://ofm.wa.gov/sites/default/files/public/legacy/researchbriefs/2013/brief070.pdf

© 2024 Rahman MS et al. *JAMA Network Open.*

eFigure 1. The Percentage of the Population According to Accessibility Level to Dental Care in 50 States and Washington, DC

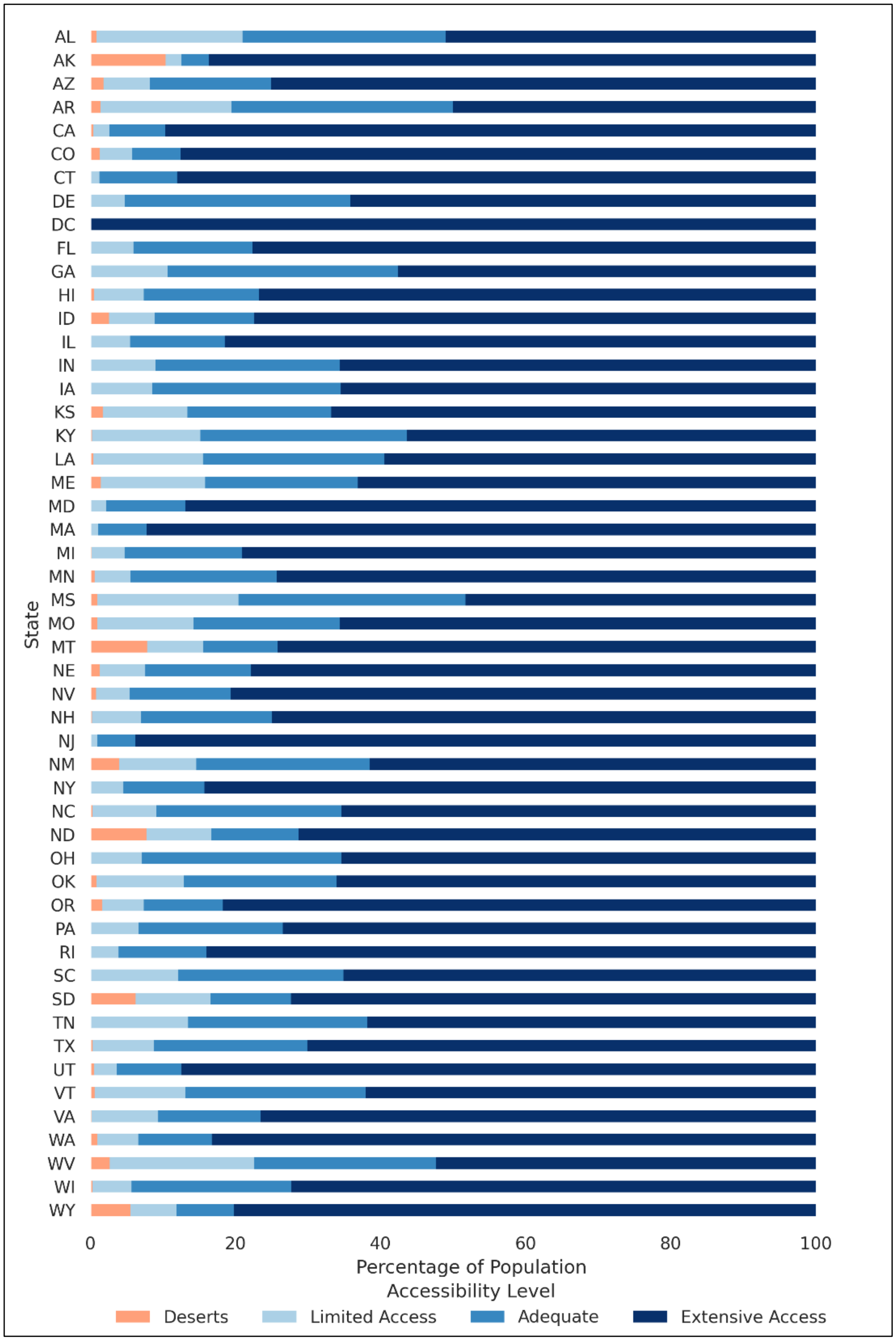

Note. Accessibility analysis considering all dentists (general clinics and specialty clinics). Accessibility analysis considered all dentists in general clinics and specialty clinics. Authors' analysis of data from 1) dentist database from IQVIA; and 2) American Community Survey (ACS) 2022 population estimates from the US Census Bureau.

© 2024 Rahman MS et al. *JAMA Network Open.*

eFigure 2. The Distribution of Accessibility Scores to Dental Care in Rural and Urban Areas

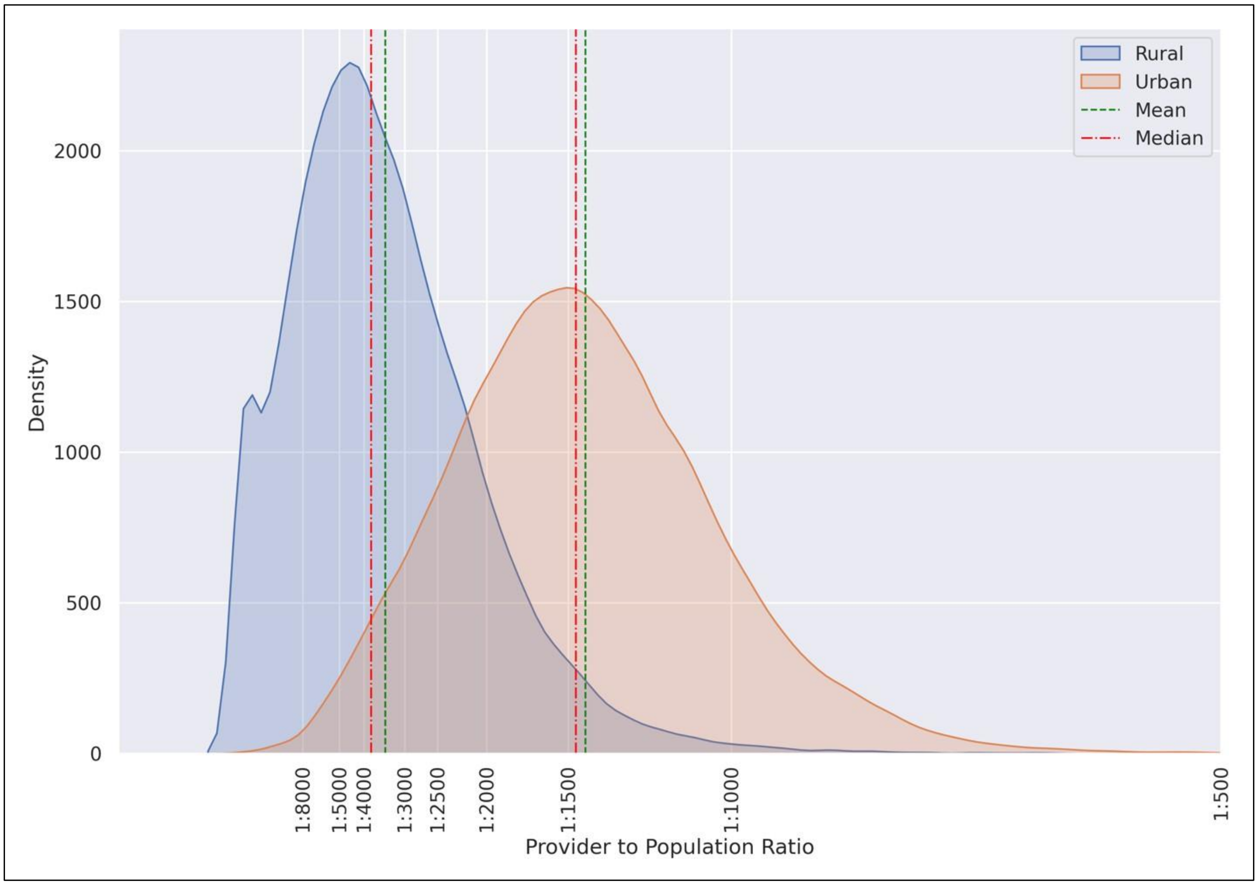

Note: Accessibility analysis considering all dentists (general clinics and specialty clinics). Authors' analysis of data from 1) dentist database from IQVIA; and 2) American Community Survey (ACS) 2022 population estimates from the US Census Bureau. Block groups are defined as rural if more than half of their population resides in rural areas.

© 2024 Rahman MS et al. *JAMA Network Open.*

eFigure 3. Dental Deserts and Dental Care Shortage Areas at the Block Group Level

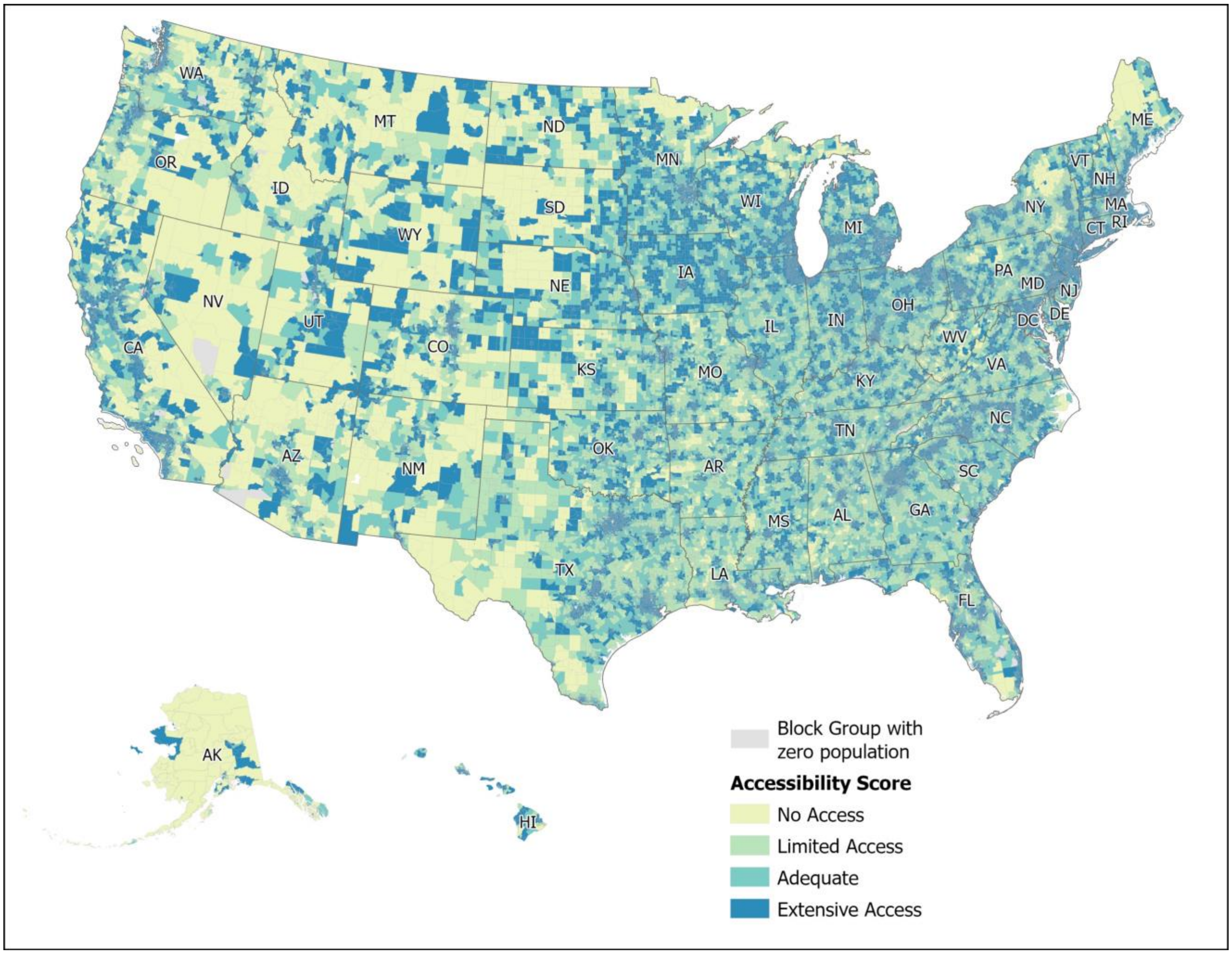

Note: Accessibility analysis considered general dentists and restricting the population to adults. Authors' analysis of data from 1) dentist database from IQVIA; 2) American Community Survey (ACS) 2022 estimates of population and TIGER /line shapefiles from US Census Bureau.

© 2024 Rahman MS et al. *JAMA Network Open.*

eFigure 4. Mean Provider-to-Population Ratio According to the Gini Coefficient of Accessibility

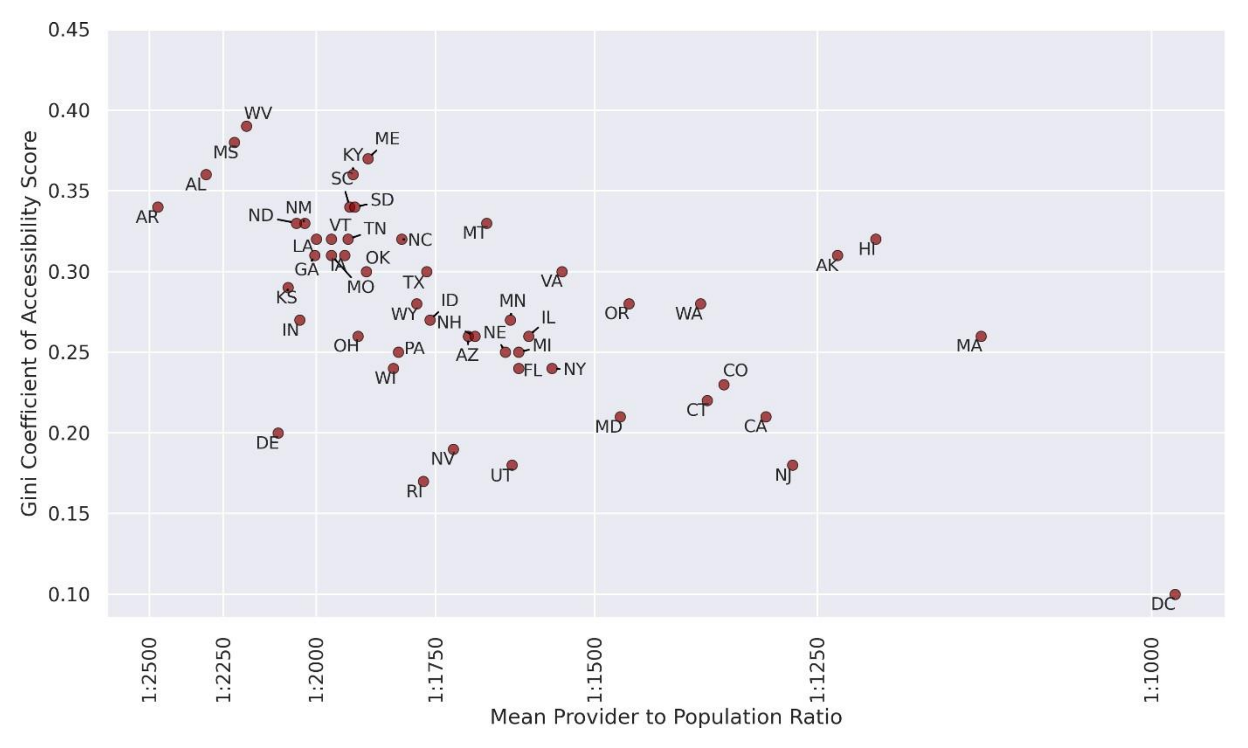

Note: Accessibility analysis considered all (general clinics and specialty clinics). Authors' analysis of data from 1) dentist database from IQVIA; and 2) American Community Survey (ACS) 2022 population estimates from US Census Bureau. The Gini coefficient of accessibility scores at the state level is calculated using accessibility scores at the block group level for each state.

© 2024 Rahman MS et al. *JAMA Network Open.*
