# Supplementary material for: Dental Clinic Deserts in the US: Spatial Accessibility Analysis
Source: JAMA Netw Open. 2024 Dec 23;7(12):e2451625. doi: 10.1001/jamanetworkopen.2024.51625 (PMC11667347; doi:10.1001/jamanetworkopen.2024.51625)
Supplement: Supplement 2. — Data Sharing Statement [file jamanetwopen-e2451625-s002.pdf]

Data Sharing Statement

Rahman. Dental Clinic Deserts in the US: Spatial Accessibility Analysis. *JAMA Netw Open*. Published December 23, 2024. doi:10.1001/jamanetworkopen.2024.51625

Data
Data available: No

Additional Information
Explanation for why data not available: We used restricted data and the Data use agreement prohibit us from publishing the data.
